# Large polarization and record-high performance of energy storage induced by a phase change in organic molecular crystals†

**DOI:** 10.1039/d1sc02729h

**Published:** 2021-10-06

**Authors:** Sachio Horiuchi, Shoji Ishibashi

**Affiliations:** Research Institute for Advanced Electronics and Photonics (RIAEP), National Institute of Advanced Industrial Science and Technology (AIST) Tsukuba Ibaraki 305-8565 Japan; Research Center for Computational Design of Advanced Functional Materials (CD-FMat), National Institute of Advanced Industrial Science and Technology (AIST) Tsukuba 305-8568 Japan

## Abstract

Dielectrics that undergo electric-field-induced phase changes are promising for use as high-power electrical energy storage materials and transducers. We demonstrate the stepwise on/off switching of large polarization in a series of dielectrics by flipping their antipolar or canted electric dipoles *via* proton transfer and inducing simultaneous geometric changes in their π-conjugation system. Among antiferroelectric organic molecular crystals, the largest-magnitude polarization jump was obtained as 18 μC cm^−2^ through revisited measurements of squaric acid (SQA) crystals with improved dielectric strength. The second-best polarization jump of 15.1 μC cm^−2^ was achieved with a newly discovered antiferroelectric, furan-3,4-dicarboxylic acid. The field-induced dielectric phase changes show rich variations in their mechanisms. The quadruple polarization hysteresis loop observed for a 3-(4-chlorophenyl)propiolic acid crystal was caused by a two-step phase transition with moderate polarization jumps. The ferroelectric 2-phenylmalondialdehyde single crystal having canted dipoles behaved as an amphoteric dielectric, exhibiting a single or double polarization hysteresis loop depending on the direction of the external field. The magnitude of a series of observed polarizations was consistently reproduced within the simplest sublattice model by the density functional theory calculations of dipole moments flipping over a hydrogen-bonded chain or sheet (sublattice) irrespective of compounds. This finding guarantees a tool that will deepen our understanding of the microscopic phase-change mechanisms and accelerate the materials design and exploration for improving energy-storage performance. The excellent energy-storage performance of SQA was demonstrated by both a high recoverable energy-storage density *W*_r_ of 3.3 J cm^−3^ and a nearly ideal efficiency (90%). Because of the low crystal density, the corresponding energy density per mass 
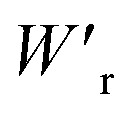
 (1.75 J g^−1^) exceeded those derived from the highest *W*_r_ values (∼8–11 J cm^−3^) reported for several bulk antiferroelectric ceramics 
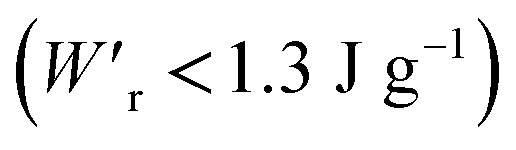
, without modification to relaxor forms.

## Abstract 2

Electric-field induced phase changes, which are promising for use in high-power electrical energy storage, can be realized in a series of organic dielectrics by flipping the antipolar or canted electric dipoles *via* proton transfer.

## Graphical abstract

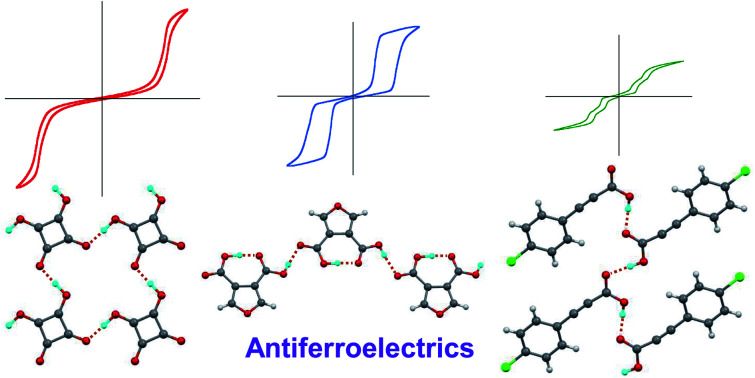

## Introduction

Highly polarizable dielectrics have been used in diverse electronic, mechatronic, and optoelectronic applications.^1,2^ In particular, phase-change dielectrics accompanied by a large polarization jump are desired for high-power electrical energy storage, which is increasingly demanded with the expansion of modern commercialization.^3,4^ One of the most suitable dielectrics is the antiferroelectric,^5,6^ in which permanent dipoles can be reversibly switched between antiparallel and parallel arrangements by changing the amplitude of an externally applied electric field.^7–11^ The reversibility between the antiferroelectric (AFE) and ferroelectric (FE) phases yields electric polarization (*P*) *vs.* electric field (*E*) hysteresis, where the *P*–*E* curves exhibit double loops instead of the single loop exhibited by ferroelectrics. The stored energy density *W*_s_ during the forward (antiferroelectric-to-ferroelectric phase) switching, the recoverable energy density *W*_r_ during the backward (ferroelectric-to-antiferroelectric AFE phase) switching, and the efficiency *η* can be evaluated through numerical integration of the *P*–*E* curves according to the following equations:1
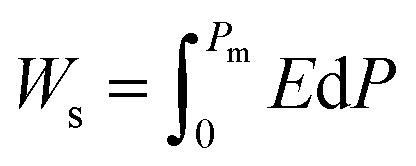
2
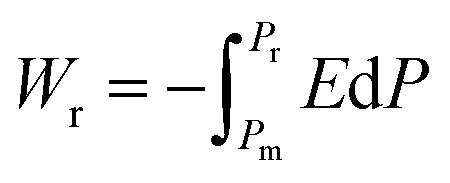

3 *η* = *W*_r_/*W*_s_

where *P*_m_ and *P*_r_ are maximum and remanent polarizations, respectively. While the voltage across conventional capacitors decreases linearly as they are discharged, the strongly nonlinear-type capacitors that exhibit the polarization jump can retain their voltage. This characteristic can simplify the electronics required to deliver a constant voltage from a capacitor. In addition, antiferroelectrics can store energy at a high density more effectively than linear dielectrics and ferroelectrics. Especially high performance has been achieved with lead-containing antiferroelectrics,^12–14^ as exemplified by (Pb,La)(Zr,Ti)O_3_ (PLZT) compounds, which have been commercially used in dc link condensers. Also, extensive research has led to remarkable improvements in the electric storage performance of lead-free alternatives.^15–18^ Ultrahigh energy storage has also been achieved by modifying these antiferroelectrics into relaxors.^19–22^

Organic molecular compounds have additional advantages when used in energy storage devices, such as mechanical flexibility, low density, and environmental benignity, as well as good dielectric strength. For instance, free-standing polycrystalline ferroelectric films have been prepared from small organic molecules.^23^ Over the past decade, we have discovered antiferroelectric switching or analogous metaelectric transitions in several hydrogen-bonded compounds.^24–27^ Highly efficient energy storage in a squaric acid (SQA) crystal, which comprises an antiparallel array of polar sheets, has been highlighted. The molecular dipole moments are reoriented through proton tautomerism (also known as prototropy), which simultaneously relocates the double bonds of a π-conjugated system and a proton of the adjacent hydrogen bond.^26^ The next challenge is to increase the stored energy density, which requires increasing the polarization jump Δ*P* and the switching field *E*_sw_.

Here, we develop a series of prototropic organic dielectrics having antipolar or canted electric dipoles. Excellent polarization performance, with a Δ*P* exceeding 15 μC cm^−2^, is achieved by improving the dielectric strength of SQA and by preparing new antiferroelectrics: deuterated SQA-*d*_2_ and furan-3,4-dicarboxylic acid (FDC). The materials development is accompanied by the discovery of multiple phase changes in another new antiferroelectric: 3-(4-chlorophenyl)propiolic acid (CPPLA). The alternative approach to antiferroelectric phase switching is to exploit the crystal anisotropy of a ferroelectric having canted dipoles. For each prototropic antiferroelectric investigated, the polarization of the polar subunit (*i.e.*, a hydrogen-bonded sheet or chain) is theoretically simulated and its simple flipping model is examined to explain the polarization jump. Record high energy-storage performances are also clarified in comparison with the corresponding performances of inorganic antiferroelectrics.

## Results and discussion

### SQA and deuterated SQA-*d*_2_

**Fig. 1:**
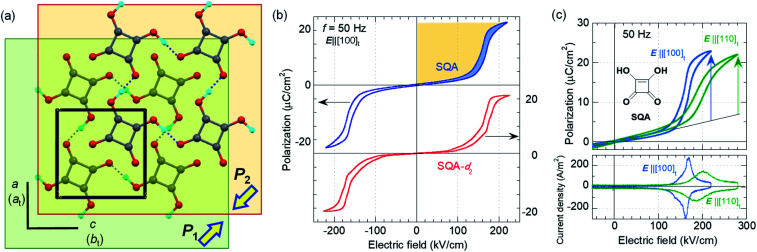
Squaric acid (SQA): (a) crystal structure viewed along the *b* (*c*_tetra_) direction. Arrows point to the directions of sheet (sublattice) polarizations ***P***_1_ and ***P***_2_. (b) Electric polarization (*P*) *vs.* electric field (*E*) hysteresis loops for SQA and its deuterated SQA-*d*_2_ crystals at room temperature. Orange and blue areas correspond to the recoverable energy density *W*_r_ (orange) and the unrecoverable energy density *W*_loss_ (blue area). (c) *P*–*E* hysteresis loops and corresponding *J*–*E* curves with ***E***‖〈100 〉_tetra_ and ***E***‖〈110〉_tetra_ configurations for an SQA crystal at room temperature and at *f* = 50 Hz.

The SQA crystal^28–30^ is a layered antiferroelectric at temperatures less than 373 K. Its structure belongs to the monoclinic *P*2_1_/*m* space group with pseudotetragonal symmetry. The two-dimensional hydrogen-bonding network constructs dipolar molecular sheets, the polarities of which alternate along the *c*_tetra_-direction. Although previous polarization hysteresis experiments^26^ showed that the best field-induced polarization was achieved at that time, their maximum field amplitude was set at ∼150 kV cm^−1^ to avoid electric breakdown of the test single crystal. Higher-quality single crystals enabled us to increase the maximum field strength (to 220–230 kV cm^−1^) in this re-examination of the SQA and to conduct new tests on the deuterated SQA-*d*_2_. In Fig. 1b and c, the entire switching process is shown together with a steep polarization jump in the corresponding *P*–*E* curve and a sharp peak in the corresponding current density (*J*)–*E* curve. With an ***E***‖[100]_tetra_ configuration at room temperature, the polarization jump Δ*P* was optimized to 17.2 and 18.4 μC cm^−2^ for the SQA and SQA-*d*_2_ crystals, respectively. These polarizations are greatly improved compared with that previously reported for SQA (10.5 μC cm^−2^)^26^ and are the largest polarizations reported for organic molecular antiferroelectric compounds.

Immediately after the discovery of antiferroelectricity, Kittel introduced the Landau-type macroscopic model comprising two interpenetrating sublattices with opposite polarizations.^7^ For the antiferroelectrics and a dipole-canted ferroelectric examined herein, the sublattices can also be defined by the periodic array of the polar subunits (chains or sheets) of identical polarities. The SQA crystal contains two sublattices with polarizations ***P***_1_ and ***P***_2_ (= −***P***_1_) being parallel to the 〈110〉_tetra_ direction. As theoretically simulated elsewhere,^31^ the external field induces a 90° rotation of ***P***_1_ or ***P***_2_, causing the polarization jump 
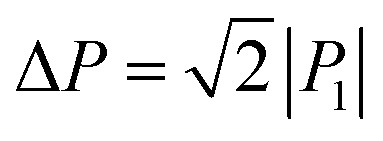
 (16.4 μC cm^−2^) along the 〈100〉_tetra_ direction, rather than a 180° flip of ***P***_1_ or ***P***_2_, which would give Δ*P* = 2|***P***_1_| (23.2 μC cm^−2^) along the 〈110〉_tetra_ direction. Notably, this interpretation does not change under this revision because the easy switching axes are 〈100〉_tetra_ and the observed polarization jump Δ*P* is very similar to 
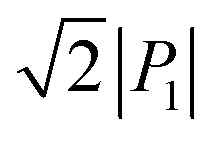
, as clearly shown in Fig. 1c.

### Furan-3,4-dicarboxylic acid (FDC) with large polarization

While the polar crystal structures of ferroelectrics usually reveal some additional hidden crystal symmetries (pseudosymmetries), similar key signatures are available for researchers searching for new antiferroelectric candidates. Here, we have discovered the new antiferroelectric furan-3,4-dicarboxylic acid (FDC) crystals, which exhibit the second-best polarization jump among organic antiferroelectrics. In the Cambridge Structure Database, all three available datasets concern the identical monoclinic polymorph (denoted as the α-form hereafter) grown from aqueous solution. In the first structural analysis (ref code: FURDCB), Williams *et al.* found that both acidic protons were disordered by crystal symmetry (space group *P*2_1_/*m*).^32^ Later, Semmingsen *et al.* redetermined the crystal structure at *T* = 125 K and found that the appearance of weak Bragg spots was caused by the antipolar arrangement of protons with twofold periodicity along the *c*-axis (ref code: FURDCB01).^33^ Our careful reassessment confirmed the validity of the latter structure even at room temperature. The *c*/2-translation symmetry is hidden in this antipolar structure. In the presence of pseudosymmetry, the antipolar and polar structures can be interconverted by rearranging the protons with minimalized modulation of the host lattice. The actual crystal structure belongs to the *P*2_1_/*c* space group (#14). The global crystal symmetry except the two protons has a mirror plane normal to the molecular plane, in addition to the aforementioned *c*/2-translation. The hydrogen-bonded molecular chains parallel to the *b*-direction have longitudinal dipoles whose polarities alternate along the *c*-direction.

Two additional polymorphs were newly generated by sublimation under reduced pressure: rhombus plates of β-FDC (major form; see ESI Fig. S1† for detailed molecular packing with orthorhombic *Pbcm* symmetry) and rectangular plates of γ-FDC (minor form; triclinic). Although both crystal forms are similar to α-FDC with respect to their hydrogen-bonded molecular sequence (Fig. S2†), polarization switching is hardly expected in the β-FDC crystal, which lacks the corresponding pseudosymmetry.

The crystal structure of γ-FDC exhibits a *C*-centered monoclinic lattice but exhibits only inversion symmetry. Instead of the unconventional space group *C*1̄, we used the equivalent triclinic space group *P*1̄ for the structural determination. The *a*/2-translation symmetry is hidden in this antipolar structure. The large unit cell of the γ-FDC crystal accommodates four sublattices ***P***_1_, ***P***_2_ (= −***P***_1_), ***P***_3_, and ***P***_4_ (= −***P***_3_). Therefore, flipping ***P***_1_ and ***P***_3_ (or ***P***_2_ and ***P***_4_) gives rise to Δ*P* = |2***P***_1_ + 2***P***_3_|.

**Fig. 2:**
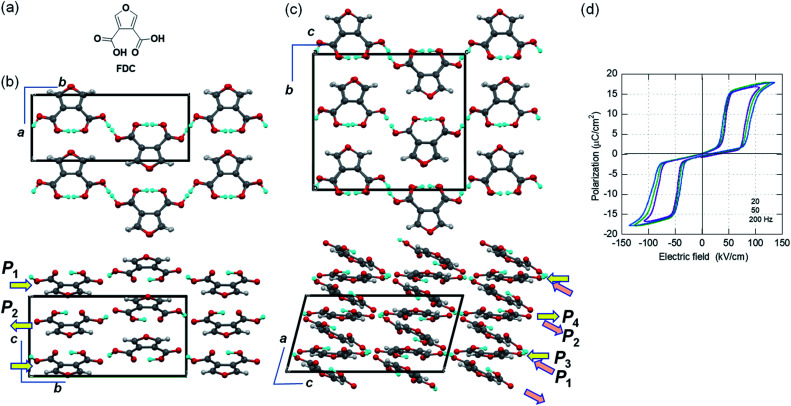
Furan-3,4-dicarboxylic acid (FDC): (a) chemical structure. (b) Molecular arrangements in the α-form crystal viewed along the stacking *c*- (top) and *a*-directions (bottom). (c) Molecular arrangements in the γ-form crystal viewed along the stacking *a*- (top) and *b*-directions (bottom). Arrows represent the polarity of the hydrogen-bonded chains. (d) *P*–*E* hysteresis loops with ***E***‖[1̄02] configurations in the γ-FDC crystal at room temperature and at various frequencies.

We demonstrated the expected phase-change phenomena described in the preceding paragraph for the γ-FDC crystal (Fig. 2d). The results of *P*–*E* hysteresis measurements with the applied ac electric field configuration *E*‖[1̄02] show the double loop characteristic of the antiferroelectric–ferroelectric transition. The observed polarization jump Δ*P*, which was as large as 15.1 μC cm^−2^, is slightly smaller than those of SQA but the second-highest among those of organic antiferroelectric crystals. The switching at ∼70 kV cm^−1^ is accompanied by a large hysteresis width of ∼50 kV cm^−1^.

Although the α-form crystal is predicted to show a similarly large Δ*P*, its small size precluded satisfactory measurements. For the β-FDC crystal, no signatures of phase-change phenomena were detected at least up to 180 kV cm^−1^ in the measurements at room temperature and at 80 °C, as suggested by the aforementioned structural assessments.

### Antiferroelectric with multiple switching processes

We discovered the new antiferroelectric CPPLA crystal while seeking for additional hidden crystal symmetries (pseudosymmetries) in the reported crystal structures. Das *et al.* reported a polar monoclinic crystal structure for the iodine-substituted derivative 3-(4-iodophenyl)propiolic acid (ref code: BEFSUB),^34^ in which we noticed pseudo-inversion symmetry, suggesting a possible ferroelectric. They also reported paraelectric-like crystal structures of CPPLA, in which the hydrogen-bonded molecular sequence pinpoints the disordered (or centered) hydrogen atom on each hydrogen bond because of inversion symmetry (ref code: SUHSET).^34,35^ In contrast, our reexamination of the single-crystal structure by X-ray diffraction revealed additional weak Bragg spots indicative of the doubled periodicity as (***a***, ***b***, ***c***) = (−***a***_p_ − ***c***_p_, −***a***_p_ + ***c***_p_, ***b***_p_). The unit-cell doubling is caused by the antipolar ordering of asymmetrically located hydrogen atoms in the revised crystal structure, which contains two sublattices with polarizations ***P***_1_ and ***P***_2_ (= −***P***_1_) and thus suggests possible antiferroelectricity along the *b*-direction.

**Fig. 3:**
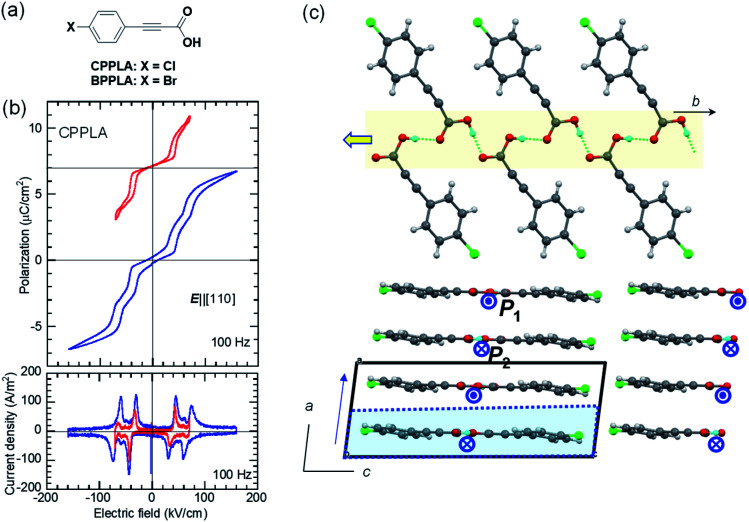
3-(4-Chlorophenyl)propiolic acid (CPPLA): (a) chemical structure. (b) *P*–*E* hysteresis loops obtained with different maximum fields at room temperature (top) and the corresponding *J*–*E* curve obtained from the quadruple hysteresis loops, indicating multiple field-induced switching (bottom). (c) A hydrogen-bonded polar molecular ribbon (top) and the crystal structure viewed along the *b*-direction (bottom). Arrows and arrowheads represent the polarity of ribbons. The dotted cell corresponds to the pseudosymmetry of *a*/2.

Consistent with this expectation, the *P*–*E* curve (top panel of Fig. 3b) shows a double hysteresis loop when the maximum field amplitude of 70 kV cm^−1^ is applied in the ***E***‖[110] configuration (instead of the ideal ***E***‖***b*** configuration because of the crystal shape). However, the application of a higher field amplitude induced an additional polarization jump, causing a quadruple polarization hysteresis loop. This behavior is the manifestation of field-induced successive phase transitions with the antiparallel dipoles flipped half-by-half. Therefore, the intermediate phase is regarded as the ferrielectric state.

### Amphoteric behavior of a dipole-canted ferroelectric

**Fig. 4:**
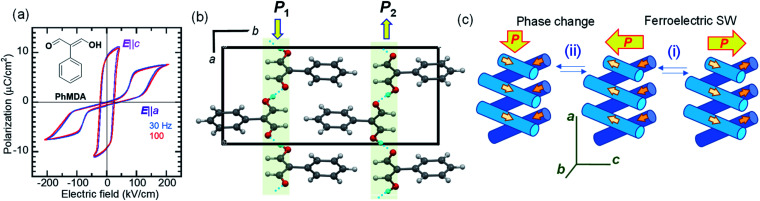
2-Phenylmalondialdehyde (PhMDA): (a) *P*–*E* hysteresis loops at room temperature, as measured with a triangular ac electric field. The applied electric field configurations of ***E***‖*a* and ***E***‖*c* are normal and parallel to the spontaneous polarization, respectively. (Inset) The chemical structure of PhMDA. (b) Hydrogen-bonded polar molecular sequences viewed along the *c*-direction. (c) Schematics of changes in sublattice polarizations (small arrows) of the hydrogen-bonded chains and the total bulk polarization (large arrows) during the (i) ferroelectric polarization reversal and (ii) field-induced phase change.

While most ferroelectric crystals have fully aligned dipoles, the ferroelectric 2-phenylmalondialdehyde (PhMDA) single crystal exhibits a canted arrangement of dipolar chains.^36^ Here, we report amphoteric behavior, where a single or double polarization hysteresis loop is obtained depending on the direction of the external field (Fig. 4a). In the orthorhombic crystal with space group *Pna*2_1_, the PhMDA molecules form polar hydrogen-bonded chains (Fig. 4b). As indicated by small open arrows in Fig. 4c, each chain has a sublattice polarization ***P***_1_ along the [102] direction or ***P***_2_ along the [1̄02] direction. Here, the crystal symmetry demands *P*_1*x*_ = –*P*_2*x*_, *P*_1*y*_ = *P*_2*y*_ = 0, *P*_1*z*_ = *P*_2*z*_; the net polarization 2|*P*_1*z*_| then emerges in the *c*-direction. Specifically, the configuration of chain dipoles is antiparallel in the *a*-direction components and parallel in the *c*-direction components. The ferroelectricity observed with the *E*‖*c* configuration corresponds to the polarization reversal induced by flipping both ***P***_1_ and ***P***_2_ according to process (i) in Fig. 4c.

In the ***E***‖***a*** configuration, the antiferroelectric-like switching with a Δ*P* of 5.8 μC cm^−2^ appears at ∼110 kV cm^−1^, which is substantially greater than the magnitude of the coercive field along the *c*-direction (∼20 kV cm^−1^). The most plausible mechanism is process (ii) in Fig. 4c. The increasing/decreasing electric field flips either ***P***_1_ or ***P***_2_, which causes the observation of Δ*P*_*x*_ = 2|*P*_1*x*_| through a 90° rotation of the spontaneous polarization from the the *c*- to the *a*-direction and *vice versa*. In this example, the antiferroelectric-like functionalities can be achieved even by rotating polarizations through the field-induced transition between two different ferroelectric phases.

### Theoretical evaluation of polarization

The experimentally optimized spontaneous polarizations of organic ferroelectrics have recently been reproduced with excellent accuracy using density functional theory (DFT) calculations and the Berry phase formalism of electric polarization.^37^ Although the corresponding theoretical evaluations are rare for organic antiferroelectrics, SQA represents an example in which the microscopic switching process has been successfully identified in terms of a sublattice polarization model through comparisons with experimental data.^31^ In the present work, the sublattice polarizations are simulated for other prototropic antiferroelectrics (FDC, CPPLA, and benzimidazoles). Except for the γ-FDC crystal, the two sublattices, which interpenetrate each other, construct the antipolar or dipole-canted structure. First, one of them is extracted as a periodic polar crystal lattice and its sublattice polarization ***P***_1_ is computed. Together with its symmetry-related sublattice polarization ***P***_2_, the theoretical polarization Δ***P***^cal^ is calculated by flipping either ***P***_1_ or ***P***_2_. For the γ-FDC crystal, two of four sublattices were extracted for calculating the crystallographically independent ***P***_1_ and ***P***_4_. The sublattice polarizations were calculated for different degrees of polar distortion *λ* between the reference symmetrized (hypothetical paraelectric, *λ* = 0) and fully polar (ferroelectric, *λ* = 1) configurations. As shown in Fig. S3,† the smooth *λ*-dependence confirms the validity of each simulation.

**Fig. 5:**
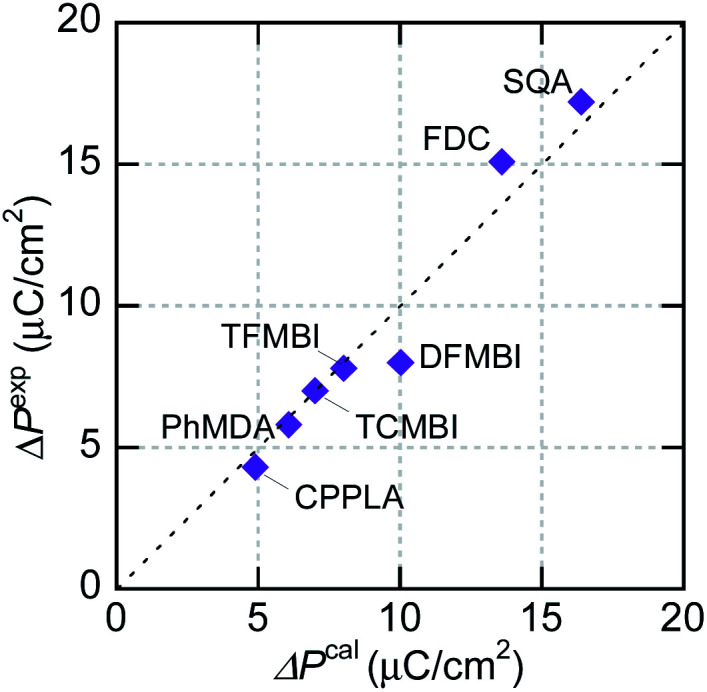
Comparison of field-induced polarizations of prototropic antiferroelectrics and an amphoteric dielectric, PhMDA. Experimentally observed polarization jump |Δ*P*^exp^| at room temperature *versus* calculated total polarization |Δ*P*^cal^| assuming a fully aligned sublattice polarization.

In the γ-FDC crystal, each hydrogen-bonded chain is highly polarized and the resultant large |Δ***P***^cal^| of 13.2 μC cm^−2^ directed along the [1̄02] is similar to the experimentally observed polarization jump |Δ***P***^exp^| (15.1 μC cm^−2^). Note that nearly the same |Δ***P***^cal^| values were computed for both the α- and β-forms, which have similar hydrogen-bonded molecular sequences (Fig. S2†). Likewise, excellent agreement between |Δ***P***^cal^| and |Δ***P***^exp^| is confirmed for a series of prototropic antiferroelectrics, as demonstrated in Fig. 5. Regarding the CPPLA crystal, the entire polarization jump of the quadruple hysteresis loops (4.3 μC cm^−2^) is explained well by the full alignment of chain polarizations (the ***E***‖[110]-direction component of Δ*P*^cal^ is 4.8 μC cm^−2^).

DFT calculations were also carried out for the dipole-canted ferroelectric PhMDA to evaluate its amphoteric behavior quantitatively. The calculated sublattice ***P***_1_ is (3.0, 0.0, 4.5) μC cm^−2^, yielding its counterpart *P*_2_ = (−3.0, 0.0, 4.5) μC cm^−2^ by symmetry. The resultant spontaneous polarization ***P***_s_ = ***P***_1_ + ***P***_2_ = (0, 0, 2|*P*_1*z*_|) is (0, 0, 9.0) μC cm^−2^ and coincides with the experimental *P*_s_ (9 μC cm^−2^) emerging in the *c*-direction. With the ***E***‖***a*** configuration, the predicted polarization jump Δ*P*_*x*_ = 2|*P*_1*x*_| = 6.1 μC cm^−2^ agrees well with the experimental Δ*P* of 5.8 μC cm^−2^.

### Energy-storage performance

**Fig. 6:**
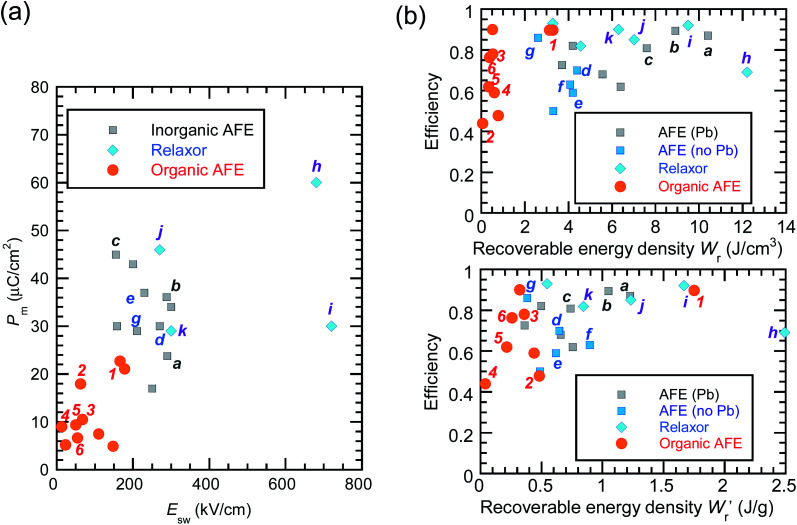
Energy-storage performance of organic molecular antiferroelectric (AFE) crystals at room temperature in comparison with reported performance for high-performing inorganic AFE or relaxor ceramics. (a) The field-induced maximum polarization *vs.* switching field. (b) The energy storage efficiency *versus* the recoverable energy density per unit volume (top) or weight (bottom). Bold alphanumeric characters represent organic antiferroelectrics (**1**, SQA; **2**, γ-FDC; **3**: DFMBI; **4**, TFMBI; **5**, TCMBI; **6**, CPPLA), inorganic antiferroelectrics (**a**, (Pb_0.98_La_0.02_)(Zr_0.55_Sn_0.45_)_0.995_O_3_;^12^**b**, (Pb_0.98_La_0.02_)(Zr_0.49_Sn_0.51_)_0.995_O_3_;^13^**c**, PbHfO_3_;^14^**d**, Ag_0.94_La_0.02_NbO_3_;^15^**e**, Ag(Nb_0.85_Ta_0.15_)O_3_;^16^**f**, 0.90(K_0.5_Na_0.5_)NbO_3_–0.10Bi(Mg_2/3_Nb_1/3_)O_3_;^17^**g**, (Ag_0.91_Bi_0.03_)NbO_3_),^18^ and inorganic antiferroelectric relaxors (**h**, 0.76NaNbO_3_–0.24(Bi_0.5_Na_0.5_)TiO_3_;^19^**i**, 0.55(Bi_0.5_Na_0.5_)TiO_3_–0.45(Sr_0.5_Bi_0.5_)TiO_3_;^20^**j**, 0.78(Bi_0.5_Na_0.5_)TiO_3_–0.22NaNbO_3_;^21^**k**, Ag(Nb_0.45_Ta_0.55_)O_3_).^22^

The *P*–*E* curves of FDC, CPPLA, and PhMDA crystals were also measured at higher temperatures (Fig. S4†). The FDC crystal revealed very weak temperature dependence up to 420 K. For CPPLA and PhMDA as well as for SQA,^26^ both the Δ*P* and switching field decrease with heating, and thus the stored energy densities are diminished from the room-temperature performance. Regarding the energy-storage performance of all of the prototropic antiferroelectrics, Table 1 summarizes the room-temperature performance obtained according to eqn (1)–(3). Fig. 6 shows a plot of these data in comparison with those of bulk antiferroelectric ceramics and their relaxor modifications, which exhibit high or ultrahigh energy-storage densities. Among the prototropic antiferroelectrics, SQA (and SQA-*d*_2_) crystals exhibit both the highest recoverable energy-storage density (*W*_r_ = 3.3 J cm^−3^) and nearly ideal efficiency (*η* = 0.90). As shown in Fig. 6a, the excellent *W*_r_ of SQA is justified by its best performance with respect to both the maximum polarization *P*_m_ and the switching field *E*_sw_ (the average of forward and backward phase switching fields). However, the *W*_r_, *P*_m_, and *E*_sw_ values are smaller than those of the bulk antiferroelectric ceramics and their relaxor modifications,^12–22,38–44^ as evident in Fig. 6a and b. The magnitude relation of the performance is drastically different in Fig. 6c, which is a replot of the data against the stored energy density per weight 
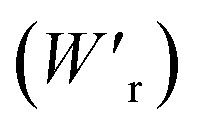
 instead of that per unit volume (*W*_r_). This difference is attributed to the crystal density *ρ* of the organic antiferroelectrics (1.3–1.9 g cm^−3^) being substantially lower than those^45^ of lead-containing (8.5–10.3 g cm^−3^) and lead-free antiferroelectrics (4.6–8.1 g cm^−3^). The 
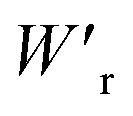
 (1.75 J g^−1^) of SQA (*ρ* = 1.88 g cm^−3^) exceeds those derived from the highest *W*_r_ values (approximately 8–11 J cm^−3^) reported for several bulk antiferroelectric ceramics 
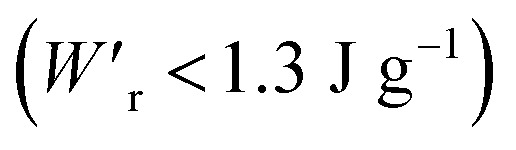
. Notably, the SQA crystal retains the highest *η* without resorting to its modification to relaxor forms. These findings are encouraging for the future applications of these materials in portable and/or mobile devices.

**Table 1:** Polarization switching and energy-storage properties of prototropic organic molecular crystals at room temperature

Compound^a^	Dipole moment of the sublattice	Polarization	Switching field	Stored energy	Efficiency	Hysteresis conditions b		
	***μ*** (D per molecule)^c^	Δ*P*^exp^ (*P*_m_) (μC cm^−2^)	*E*_sw_ (Δ*E*_sw_) (kV cm^−1^)	*W*_r_ (*W*_s_) (J cm^−3^)	*η*	*f* (Hz)	*E*_m_ (kV cm^−1^)	***E***-direction
SQA	(4.37, 0.00, 4.93)	17.2 (22.7)	166 (6)	3.29 (3.67)	0.90	50	210	‖〈100〉_tetra_
SQA^d^		10.5 (13.3)	124 (4.6)	1.44 (1.53)	0.94	100	151	‖〈100〉_tetra_
SQA-*d*_2_		18.4 (21.1)	178 (20)	3.13 (3.49)	0.90	50	225	‖〈100〉_tetra_
α-FDC	(0.0, 6.58, 0.00)	—	—	—	—	—	—	—
γ-FDC	*μ*_A_ = (1.73, 0.00, −6.29)	15.1 (17.9)	62 (40)	0.78 (1.63)	0.48	50	127	[1̄02]
	*μ*_B_ = (1.76, 0.00, 6.36)							
DFMBI^e^	(0.04, 0.00, −5.72)	8.0 (10.5)	67 (12)	0.52 (0.67)	0.78	2	86	‖[001]
TFMBI^e^	(0.36, −0.01, −5.04)	7.8 (9.0)	13 (8)	0.060 (0.137)	0.44	0.2	23	‖[001]
TCMBI^e^	(−5.31, 0.04, 0.00)	7.0 (9.4)	49 (22)	0.33 (0.54)	0.62	10	80	‖[100]
CPPLA	(1.43, 3.11, 0.29)	4.3 (6.7)	55	0.38 (0.50)	0.76	100	160	‖[110]
PhMDA	(3.33, 0.00, 4.95)	5.8 (7.5)	110 (59)	0.59 (1.00)	0.59	100	200	‖[100]

a TFMBI = 2-trifluoromethylbenzimidazole, DFMBI = 2-difluoromethylbenzimidazole, TCMBI = 2-trichloromethylbenzimidazole.

b *f* = applied frequency of triangular waves, *E*_m_ = maximum field amplitude applied.

c Dipole moment *μ*_i_ was calculated from theoretical sublattice polarization *P*_i_.

d The data from ref. 26 for SQA.

e The data in ref. 24 for three benzimidazoles was used in the analysis.

## Conclusions

Various dielectric phase-change phenomena have been demonstrated *via* studies on revisited SQA, newly developed antiferroelectrics, and the “amphoteric dielectric” PhMDA. In terms of the magnitude of the field-induced polarization jump, the best (∼18 μC cm^−2^) and second-best performances (∼15 μC cm^−2^) among organic antiferroelectrics were achieved. In particular, the improvement of the dielectric strength of SQA resulted in excellent energy-storage performance, including a high recoverable energy-storage density (*W*_r_ = 3.3 J cm^−3^), while maintaining nearly ideal efficiency (*η* = 90%). The advantage of organic molecular systems is their low crystal density, which resulted in corresponding energy densities per mass as high as 
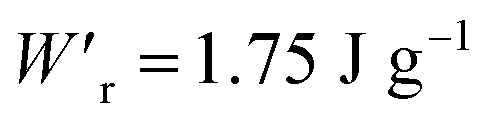
, which exceeds those derived from the highest *W*_r_ values (∼8–11 J cm^−3^) reported for several bulk antiferroelectric ceramics 
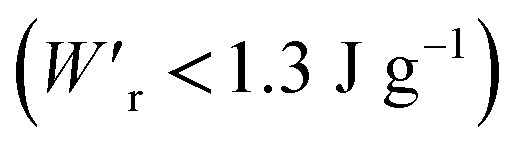
. Notably, the present SQA crystal has not yet been modified into relaxor forms.

In addition to the antiferroelectrics exhibiting conventional double hysteresis loops, the CPPLA crystal was found to exhibit quadruple polarization hysteresis loops caused by the two-step phase transition with moderate polarization jumps. The ferroelectric PhMDA single crystal represents a novel “amphoteric dielectric” that can exhibit a single or double polarization hysteresis loop depending on the direction of the applied external field relative to the directions of its canted dipole moments. Irrespective of the details of their variation, all of the observed polarizations are consistently explained by the DFT calculations combined with the simplest sublattice model. Such a theoretically precise prediction ability provides a powerful tool for improving the energy-storage performance; it will accelerate the materials design and exploration as well as deepening our understanding of the microscopic phase-change mechanisms.

## Experimental section

### Preparation and electric measurements

Commercially available SQA, FDC, PhMDA, and CPPLA were purified by repeated recrystallizations and/or temperature-gradient vacuum sublimation. The temperature-gradient sublimation under reduced pressure gave single crystals with shapes of elongated rectangular plates in the case of FDC, parallelogram-shaped plates in the case of CPPLA, and thick plates in the case of PhMDA. The bipyramidal crystals of SQA and SQA-*d*_2_, which were grown by recrystallization from hot deionized water and 99.5% D_2_O, respectively, were cut with a blade for electric measurements according to previously reported procedures.^26^ All of the electric measurements were conducted using single crystals with painted silver electrodes. The *P*–*E* hysteresis measurements were performed by applying a high-voltage triangular wave field and various alternating frequencies to single crystals, which were immersed in silicone oil to prevent atmospheric discharge. The system used to evaluate ferroelectrics (Toyo Corporation, FCE-1) comprised a current/charge–voltage converter (model 6252), an arbitrary waveform generator (Biomation 2414B), an analog-to-digital converter (WaveBook 516), and a voltage amplifier (NF Corporation, HVA4321).

### Crystallographic studies

The crystallographic data and experimental details are summarized in Table S1.† X-ray diffraction data were collected from single crystals at room temperature using graphite-monochromated Mo Kα radiation (*λ* = 0.7107 Å) and a four-circle diffractometer equipped with a two-dimensional detector [hybrid pixel detector (Rigaku AFC10 with PILATUS200K)]. CrystalStructure crystallographic software packages [Molecular Structure Corp. (MSC; Woodlands, TX) and Rigaku Corp. (Tokyo)] were used for the direct method and for the refinement of the structures. Final refinements of the nonhydrogen atoms were performed with anisotropic thermal factors. The hydrogen-bonded hydrogen atoms were found by differential Fourier synthesis and were refined isotropically; the remaining hydrogen atoms were calculated in their ideal geometrical positions.

### Theoretical calculations

First, for the experimentally obtained AFE structures, hydrogen positions were computationally optimized to minimize the total energy. Next, for each system, all of the atoms except for one polar subunit were removed from the unit cell (*λ* = 1). Reference nonpolar structures (*λ* = 0) were constructed by symmetrization. The polarization as a function of *λ* was calculated using the Berry phase approach.^46,47^ All the calculations were performed using the QMAS code^48^ based on the projector augmented-wave method^49^ and the plane-wave basis set. To describe the electronic exchange–correlation energy, the Perdew–Burke–Ernzerhof (PBE) version of the generalized gradient approximation (GGA)^50^ was used.

## Author contributions

S. H. prepared the purified single crystals, performed the dielectric measurements, conceived the study design, and wrote most of the paper. S. I performed the theoretical calculations.

## Conflicts of interest

There are no conflicts to declare.

## Supplementary Material

SC-012-D1SC02729H-s001

SC-012-D1SC02729H-s002

## References

[cit1] Lines M. E. and Glass A. M., Principles and Applications of Ferroelectrics and Related Materials, Oxford University Press, New York, 1977

[cit2] Uchino K. and Giniewicz J. R., Micromechatronics, Marcel Dekker, New York, 2003

[cit3] Yao Z., Song Z., Hao H., Yu Z., Cao M., Zhang S., Lanagan M. T., Liu H. (2017). Adv. Mater..

[cit4] Palneedi H., Peddigari M., Hwang G.-T., Jeong D.-Y., Ryu J. (2018). Adv. Funct. Mater..

[cit5] Liu Z., Lu T., Ye J., Wang G., Dong X., Withers R., Liu Y. (2018). Adv. Mater. Tech..

[cit6] Yang L., Kong X., Li F., Hao H., Cheng Z., Liu H., Li J.-F., Zhang S. (2019). Prog. Mater. Sci..

[cit7] Kittel C. (1951). Phys. Rev..

[cit8] Sawaguchi E., Maniwa H., Hoshino S. (1951). Phys. Rev..

[cit9] Shirane G., Sawaguchi E., Takagi Y. (1951). Phys. Rev..

[cit10] Tan X., Ma C., Frederick J., Beckman S., Webber K. G. (2011). J. Am. Ceram. Soc..

[cit11] Hao X., Zhai J., Kong L. B., Xu Z. (2014). Prog. Mater. Sci..

[cit12] Wang H., Liu Y., Yang T., Zhang S. (2019). Adv. Funct. Mater..

[cit13] Liu Y., Liu S., Yang T., Wang H. (2021). J. Mater. Sci..

[cit14] Wei J., Yang T., Wang H. (2019). J. Eur. Ceram. Soc..

[cit15] Gao J., Zhang Y. C., Zhao L., Lee K. Y., Liu Q., Studer A., Hinterstein M., Zhang S. J., Li J. F. (2019). J. Mater. Chem. A.

[cit16] Zhao L., Liu Q., Gao J., Zhang S., Li J. F. (2017). Adv. Mater..

[cit17] Shao T. Q., Du H. L., Ma H., Qu S. B., Wang J., Wang J. F., Wei W. Y., Xu Z. (2017). J. Mater. Chem. A.

[cit18] Tian Y., Jin L., Zhang H. F., Xu Z., Wei X. Y., Viola G. (2017). J. Mater. Chem. A.

[cit19] Qi H., Zuo R., Xie A., Tian A., Fu J., Zhang Y., Zhang S. (2019). Adv. Funct. Mater..

[cit20] Li J., Li F., Xu Z., Zhang X. (2018). Adv. Mater..

[cit21] Zou K., Dan Y., Xu H., Zhang Q., Lu Y., Huang H., He Y. (2019). Mater. Res. Bull..

[cit22] Luo N., Han K., Cabral M. J., Liao X., Zhang S., Liao C., Zhang G., Chen X., Feng Q., Li J. F., Wei Y. (2020). Nat. Commun..

[cit23] Harada J. (2021). APL Mater..

[cit24] Horiuchi S., Kagawa F., Hatahara K., Kobayashi K., Kumai R., Murakami Y., Tokura Y. (2012). Nat. Commun..

[cit25] Kobayashi K., Horiuchi S., Ishibashi S., Murakami R., Kumai R. (2018). J. Am. Chem. Soc..

[cit26] Horiuchi S., Kumai R., Ishibashi S. (2018). Chem. Sci..

[cit27] Horiuchi S., Ishibashi S., Haruki R., Kumai R., Inada S., Aoyagi S. (2020). Chem. Sci..

[cit28] Semmingsen D. (1973). Acta Chem. Scand..

[cit29] Feder J., in, Oxocarbons, ed. R. West, Academic Press, New York, 1980, pp. 141–167

[cit30] Moritomo Y., Tokura Y., Takahashi H., Mori Y. (1991). Phys. Rev. Lett..

[cit31] Ishibashi S., Horiuchi S., Kumai R. (2018). Phys. Rev. B.

[cit32] Williams D. E., Rundle R. E. (1964). J. Am. Chem. Soc..

[cit33] Semmingsen D., Nordenson S., Aasen A. (1986). Acta Chem. Scand., Ser. A.

[cit34] Das D., Jetti R. K. R., Boese R., Desiraju G. R. (2003). Cryst. Growth Des..

[cit35] Desiraju G. R., Murty B. N., Kishan K. V. R. (1990). Chem. Mater..

[cit36] Horiuchi S., Kumai R., Tokura Y. (2011). Adv. Mater..

[cit37] Horiuchi S., Kobayashi K., Kumai R., Ishibashi S. (2017). Nat. Commun..

[cit38] Zhang Q., Tong H., Chen J., Lu Y., Yang T., Yao X., He Y. (2016). Appl. Phys. Lett..

[cit39] Shen J., Wang X., Yang T., Wang H., Wei J. (2017). J. Alloys Compd..

[cit40] Zhang L., Jiang S., Fan B., Zhang G. (2015). J. Alloys Compd..

[cit41] Gao P., Liu Z., Zhang N., Wu H., Bokov A. A., Ren W., Ye Z.-G. (2019). Chem. Mater..

[cit42] Zhao L., Gao J., Liu Q., Zhang S., Li J. F. (2018). ACS Appl. Mater. Interfaces.

[cit43] Tian Y., Jin L., Zhang H., Xu Z., Wei X., Politova E., Stefanovich S. Y., Tarakina N. V., Abrahams I., Yan H. (2016). J. Mater. Chem. A.

[cit44] Li Q., Zhou C., Xu J., Yang L., Zhang X., Zeng W., Yuan C., Chen G., Rao G. (2016). J. Mater. Sci.: Mater. Electron..

[cit45] Landolt-Börnstein Numerical Data and Functional Relationships in Science and Technology, New Series, Group III: Crystal and Solid State Physics, Ferroelectric and Related Substances, ed. Y. Shiozaki, E. Nakamura and T. Mitsui, Springer-Verlag, Berlin, 2006, vol. 36

[cit46] Resta R. (1994). Rev. Mod. Phys..

[cit47] King-Smith R. D., Vanderbilt D. (1993). Phys. Rev. B: Condens. Matter Mater. Phys..

[cit48] Ishibashi S., Tamura T., Tanaka S., Kohyama M., Terakura K. (2007). Phys. Rev. B: Condens. Matter Mater. Phys..

[cit49] Blöchl P. E. (1994). Phys. Rev. B: Condens. Matter Mater. Phys..

[cit50] Perdew J. P., Burke K., Ernzerhof M. (1996). Phys. Rev. Lett..

