# Supplementary material for: Large polarization and record-high performance of energy storage induced by a phase change in organic molecular crystals
Source: Chem Sci. 2021 Oct 6;12(42):14198–206. doi: 10.1039/d1sc02729h (PMC8565377; doi:10.1039/d1sc02729h)
Supplement: SC-012-D1SC02729H-s001 [file SC-012-D1SC02729H-s001.pdf]

Electronic Supplementary Material (ESI) for Chemical Science.
This journal is © The Royal Society of Chemistry 2021

Large Polarization and Record-High Performance of Energy-Storage Induced by a Phase Change in Organic Molecular Crystals

Sachio Horiuchi[1] and Shoji Ishibashi[2]

[1]*Research Institute for Advanced Electronics and Photonics (RIAEP), National Institute of Advanced Industrial Science and Technology (AIST), AIST Tsukuba Central 5, 1-1-1 Higashi, Tsukuba, Ibaraki 305-8565, Japan*

[2]*Research Center for Computational Design of Advanced Functional Materials (CD-FMat), National Institute of Advanced Industrial Science and Technology (AIST), AIST Tsukuba Central 2, Tsukuba 305-8568, Ibaraki, Japan*

Electronic Supplementary Information

Contents

1. Structural properties
2. Theoretical calculations
3. Temperature dependence of P-E loops

1. Structural properties

Supplementary Table S1: Crystal Data and Experimental Details of FDC and CPPLA Crystals

	α-FDC	β-FDC	γ-FDC	CPPLA
Chemical formula	$C_6H_4O_5$	$C_6H_4O_5$	$C_6H_4O_5$	$C_9H_5ClO_2$
Formula wt.	156.09	156.09	156.09	180.59
Temperature (K)	296	296	296	295
a (Å)	6.0486(3)	6.8922(4)	7.3918(2)	7.1124(2)
b (Å)	14.4096(6)	14.3401(7)	12.7404(3)	7.3896(2)
c (Å)	7.3391(4)	6.3738(4)	14.4174(4)	17.2936(5)
α (deg.)	90	90	86.877(2)	79.979(2)
β (deg.)	92.538(4)	90	75.177(2)	79.262(2)
γ (deg.)	90	90	77.696(2)	65.828(3)
V (Å^3)	639.03(5)	629.95(6)	1282.42(6)	809.84(4)
Crystal system	monoclinic	orthorhombic	triclinic	triclinic
Space group	$P2_1/c$ (#2)	*Pbcm* (#57)	*P*-1(#2)	*P*-1(#2)
ρ_{calc} (g/cm^3)	1.622	1.646	1.617	1.481
Z	4	4	8	4
Dimensions (mm)	0.35×0.28×0.15	0.35×0.35×0.23	0.48×0.35×0.15	0.40×0.15×0.15
Radiation	MoKα (λ = 0.7107 Å)	MoKα (λ = 0.7107 Å)	MoKα (λ = 0.7107 Å)	MoKα (λ = 0.7107 Å)
$2\theta_{max}$ (deg.)	55	55	55	55
R_{int}	0.009	0.007	0.014	0.013
Reflection used ($2\sigma(I) < I$)	1459	775	5848	3714
No. of variables	109	73	429	225
R	0.035	0.037	0.064	0.034
wR	0.123	0.109	0.162	0.119
GOF	1.09	1.08	1.17	1.11

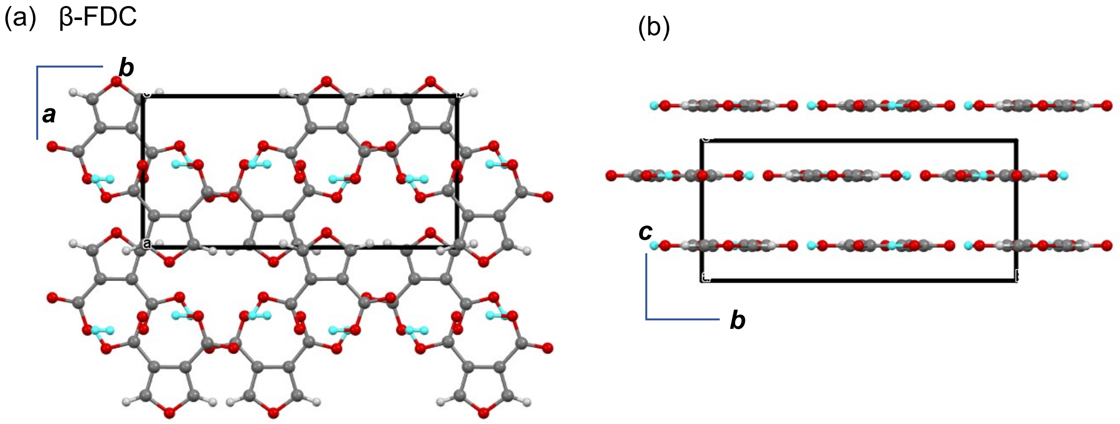

Figure S1. The crystal structure of β-FDC. Molecular arrangement viewed along the crystal (a) *c*- and (b) *a*-directions.

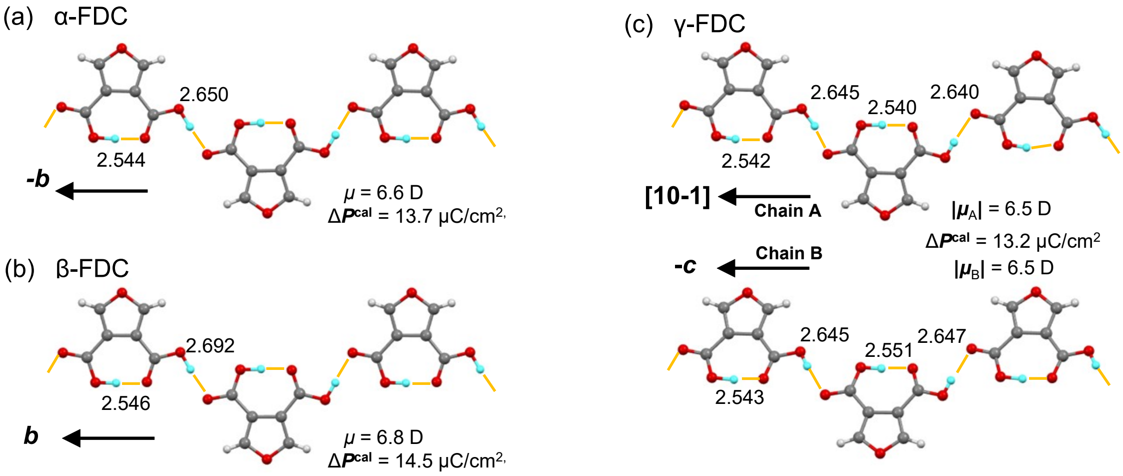

□

□**Figure S2.** Hydrogen-bonded molecular sequences in three polymorphs of FDC together with the crystallographically independent O…O distances (in Å) and calculated sublattice polarizations: (a) α-FDC; (b) β-FDC; (c) γ-FDC. The arrows indicate the polarity.

2. Theoretical calculations

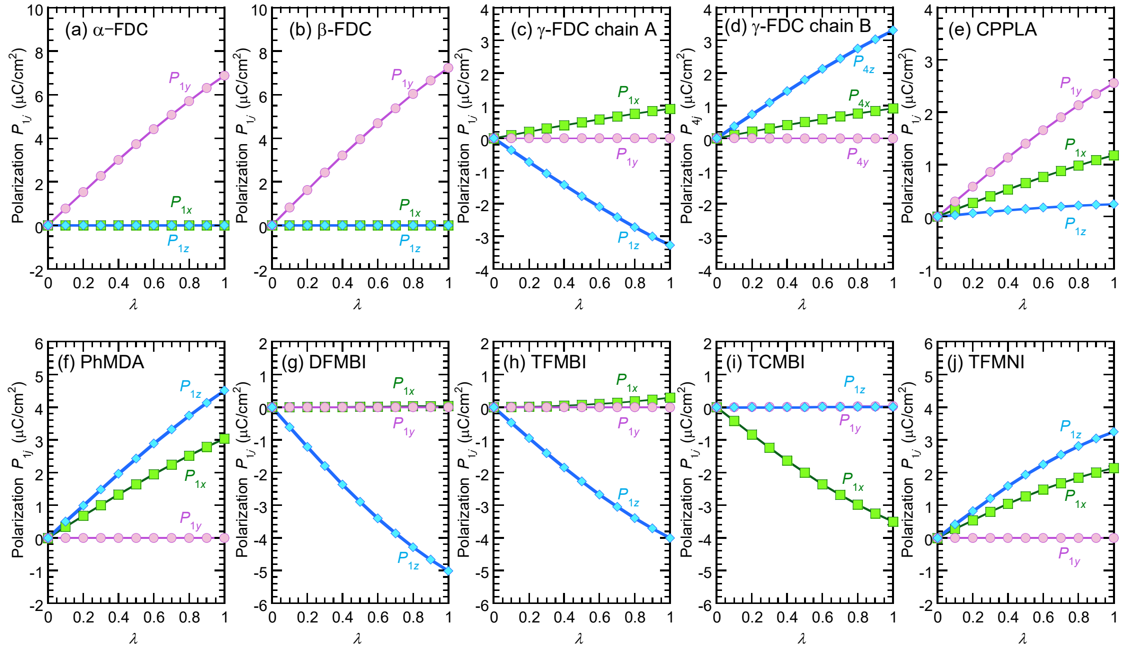

Figure S3. Evolution of the sublattice (chain) polarizations as a function of the degree of polar distortion λ ranging from the symmetrized reference (hypothetical paraelectric, λ = 0) to the fully polarized (ferroelectric, λ = 1) configuration. The Cartesian coordinate system (x, y, z) was chosen to be parallel to the crystallographic (***a***, ***b***′, ***c****) axes (***b***′ = ***c**** × ***a***) for the triclinic γ-FDC and CPPLA crystals, the (***a***, ***b***, ***c****) axes for the monoclinic α-FDC and TFMNI crystals, and the (***a****, ***b***, ***c***) axes for the monoclinic DFMBI crystal.

3. Temperature-dependent *P-E* loops

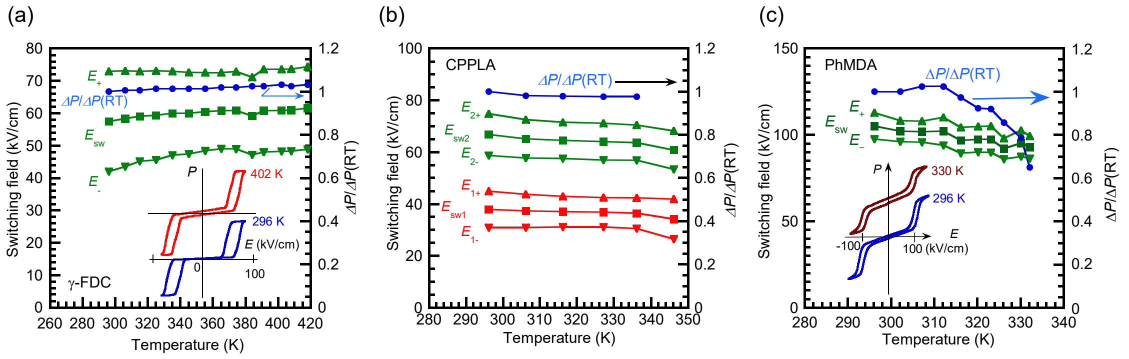

Figure S4. Temperature variations of forward (E_+), backward (E_-), and average (E_{sw}) switching fields together with the that of polarization jumps normalized at room temperature. Inset draws the *P-E* hysteresis curves at room and high temperatures. (a) γ-FDC crystal (different specimen from Fig. 2d) measured with E||[**Error!**02] configuration at 30 Hz (b) CPPLA crystal with E||[110] configuration at 100 Hz. (c) PhMDA crystal (different specimen from Fig. 4a) with E||[100] configuration at 10 Hz.
